# 626. A Mixed-methods Photographic Analysis of Cookstove Arrangements in Rural, Urban, and Slum Communities of Nepal

**DOI:** 10.1093/jbcr/irag033.452

**Published:** 2026-04-06

**Authors:** Anusha Jayaram, Dinasha D Navindi, Aldina Mesic, Pariwesh Raj Bista, Padma Maharjan, Krishna Shrestha, Manish K Yadav, Shankar Man Rai, Kiran K Nakarmi, Barclay T Stewart

**Affiliations:** Department of Surgery, Beth Israel Deaconess Medical Center / Division of Global Health, University of Washington, Boston, MA; Kaiser Permanente Los Angeles Medical Center, Los Angeles, CA; Imperial College London, London, England; University of Washington, Bhaktapur, Bagmati; Department of Burns, Plastic, & Reconstructive Surgery, Kirtipur Hospital, Kathmandu, Bagmati; Department of Burns, Plastic, & Reconstructive Surgery, Kirtipur Hospital, Kathmandu, Bagmati; Department of Burns, Plastic, & Reconstructive Surgery, Kirtipur Hospital, Kathmandu, Bagmati; Department of Burns, Plastic, & Reconstructive Surgery, Kirtipur Hospital, Kathmandu, Bagmati; Department of Burns, Plastic, & Reconstructive Surgery, Kirtipur Hospital, Kathmandu, Bagmati; Department of Surgery, University of Washington School of Medicine / Harborview Injury Prevention and Research Center, Seattle, WA

## Abstract

**Introduction:**

In Nepal, cooking is a leading cause of burn injuries, driven by unsafe stove designs, hazardous environments, reliance on biomass fuels, and limited prevention efforts. To understand the cooking environment and identify hazards for burn injuries, we conducted a photographic analysis of cookstove arrangements to generate evidence for context-specific and effective prevention strategies.

**Methods:**

We performed a mixed-methods photographic analysis of cookstove arrangements in Nepal. Photographic data were from a larger population-proportional, cluster randomized, household survey conducted in rural, urban, and slum communities. A structured visual analysis adapted from the Gallagher Cookstove Assessment was performed to assess the safety characteristics of cookstove arrangements across community types.

**Results:**

A total of 412 household cookstove arrangements were assessed individually for structural and safety features. Most rural households (30.2%) used traditional stoves such as open-fire, three-stone, or mud designs, followed by “improved” mud stoves (26.8%). Slum households used liquefied petroleum gas (LPG) stoves (79.8%), while urban households used both LPG (56.4%) and traditional designs (22.5%). Open-flame cooking was equally common in rural (5.4%) and urban (5.0%) households.

Across all communities, 72.6% of cookstove arrangements were judged high-risk for burn injury, with hazards including flames extending beyond combustion chambers, unstable stands, lack of protective barriers, and proximity to flammable materials. Combustible items (e.g., textiles or fuel containers) within 3 feet of the stove were identified in 51.5% of households. Rural communities exhibited the highest proportion of unsafe cookstove arrangements (64.7%) compared to urban (57.7%) and slum communities (49.5%) (*p*=.048). Unsafe arrangements included use of more than one stove type simultaneously, use of improvised components (e.g., non-standardized gas tubing, stove stands, pot rings), low-quality regulators on LPG stoves (e.g., lack of automatic or excess flow shut-off valves, lack of clear on–off labels, plastic fittings), and uninsulated hot contact surfaces. Only 4.4% of households had fire extinguishers within reach of the stove.

**Conclusions:**

This photographic analysis reveals substantial heterogeneity in stove type and configuration across Nepalese communities, with rural households disproportionately exposed to burn injury risk due to reliance on traditional and improvised stoves in hazardous environments. While LPG use predominates in slums and mixed strategies characterize urban areas, unsafe practices and environments are widespread across all settings.

**Applicability of Research to Practice:**

Efforts to mitigate burn injury risk in Nepal should prioritize safer stove design, enforcement of safety standards, community education, and low-cost improvements in household infrastructure.

**Funding for the study:**

Research reported in this publication was supported by the Fogarty International Center of the National Institutes of Health under grant #D43TW009345.

